# Suppression of Microbial Metabolic Pathways Inhibits the Generation of the Human Body Odor Component Diacetyl by *Staphylococcus* spp

**DOI:** 10.1371/journal.pone.0111833

**Published:** 2014-11-12

**Authors:** Takeshi Hara, Hiroshi Matsui, Hironori Shimizu

**Affiliations:** Technical Development Center, Mandom Corp., Osaka, Japan; Laurentian University, Canada

## Abstract

Diacetyl (2,3-butanedione) is a key contributor to unpleasant odors emanating from the axillae, feet, and head regions. To investigate the mechanism of diacetyl generation on human skin, resident skin bacteria were tested for the ability to produce diacetyl via metabolism of the main organic acids contained in human sweat. l-Lactate metabolism by *Staphylococcus aureus* and *Staphylococcus epidermidis* produced the highest amounts of diacetyl, as measured by high-performance liquid chromatography. *Glycyrrhiza glabra* root extract (GGR) and α-tocopheryl-l-ascorbate-2-O-phosphate diester potassium salt (EPC-K1), a phosphate diester of α-tocopherol and ascorbic acid, effectively inhibited diacetyl formation without bactericidal effects. Moreover, a metabolic flux analysis revealed that GGR and EPC-K1 suppressed diacetyl formation by inhibiting extracellular bacterial conversion of l-lactate to pyruvate or by altering intracellular metabolic flow into the citrate cycle, respectively, highlighting fundamentally distinct mechanisms by GGR and EPC-K1 to suppress diacetyl formation. These results provide new insight into diacetyl metabolism by human skin bacteria and identify a regulatory mechanism of diacetyl formation that can facilitate the development of effective deodorant agents.

## Introduction

Human body odor can be attributed to the bacterial metabolism of odorless compounds secreted from the eccrine, apocrine, and sebaceous glands. Secretions from different body parts–such as the axillae, feet, and scalp–have distinct odors [1]–[4]. In modern cultures, body odor can have a significant negative impact on the social life of individuals; the deodorant industry therefore invests considerable effort into creating technologically superior products for minimizing body odor. Many deodorant manufacturers claim that their products are specifically designed to prevent human body odor and stress sweating for as long as possible [5]. For the effective control of body odor, the characterization of different odor-causing elements–including the responsible bacteria and their metabolites–as well as the analysis of active deodorant components are essential. An understanding of how body odor is produced from complex bacterial metabolic processes is also of great interest to biologists.

(*E*)-3-Methyl-2-hexenoic acid (3M2H) is one of the major components of axillary odor [6]; a chemically related acid, 3-hydroxy-3-methylhexanoic acid (HMHA), and volatile sulfur compounds, including 3-methyl-3-sulfanylhexan-1-ol (3M3SH), are key contributors to spicy and sulfur-like odors [7]–[10]. Both 3M2H and HMHA are produced by the cleavage of 3M2H- and HMHA-glutamine by N-acyl-aminoacylase of *Corynebacterium* sp. [7], and a 3M3SH precursor has been identified as a cysteine-glycine dipeptide conjugate [11]. Many odorous steroids, particularly the 16-androstenes, 5α-androstenol, and 5α-androstenone, have been identified, and their precursors and metabolic pathways in bacteria have been well studied [12], [13]. Human feet that produce a strong odor have significantly higher bacterial population densities than those that are only mildly odorous [14], which can be ascribed to isovaleric acid [15], a *Staphylococcus epidermidis* leucine metabolite that can be inhibited by fragrant agents, including citral, citronellal, and geraniol [16]. Recently, diacetyl (2,3-butanedione) was identified as a key contributor to unpleasant odors emanating from the head region of middle-aged Japanese males [17]. We have also shown that diacetyl imparts an acid-like quality to axillary and foot odor that is distinct from that of acetic acid, propionic acid, and isovaleric acid previously reported [18].

Diacetyl is found in alcoholic beverages and dairy products, and contributes to both positive and negative sensory impressions of food flavor and taste [19], [20]. The biological mechanisms involved in diacetyl formation have been well studied, especially with respect to its regulation in yeast or lactic acid bacteria [20]–[22]. These microorganisms metabolize pyruvate and form the unstable intermediate α-acetolactate, which is then converted non-enzymatically to diacetyl by oxidative decarboxylation. Diacetyl has an extremely low olfactory threshold value that is 100 times lower than that of acetic acid, a well-known body odor compound with a hint of acidity. However, there have been no studies on how the metabolic routes responsible for diacetyl formation could be used to suppress this process on human skin.

In this study, we investigated the major human resident skin bacteria and sweat components associated with diacetyl generation, and their potential regulation by cosmetic ingredients. An *in vitro* screen identified two compounds that strongly inhibit diacetyl production, while results of a metabolic flux analysis indicated that the underlying inhibitory mechanisms of these compounds were not bactericidal, but instead affected metabolic pathways. These findings will be useful in the design of novel and effective deodorant products.

## Materials and Methods

### General

All chemicals were purchased from Wako Pure Chemical Industries, Ltd. (Osaka, Japan), unless otherwise indicated. Plant extracts were obtained from Maruzen Pharmaceutical Co., Ltd. (Hiroshima, Japan) and Ichimaru Pharcos Co., Ltd. (Gifu, Japan).

### Bacterial strains and growth conditions

*Staphylococcus aureus* NBRC13276, *Staphylococcus epidermidis* IAM1296, *Staphylococcus capitis* ATCC27843, *Staphylococcus hominis* ATCC35982, *Staphylococcus haemolyticus* ATCC29970, *Corynebacterium jeikeium* ATCC43734, *Corynebacterium xerosis* NBRC12684, *Corynebacterium minutissimum* ATCC23348, and *Corynebacterium striatum* ATCC6940, which were used as representative skin bacteria, were precultured aerobically in 9 ml soybean casein digest broth (Nihon Pharmaceutical, Tokyo, Japan) or brain heart infusion broth (Nissui Pharmaceutical, Tokyo, Japan) at 36°C for 19 h in glass tubes. Cells were collected, washed with 0.85% (w/v) saline, and resuspended to a concentration of ∼10^8^ CFU/ml in semi-synthetic medium (pH 6.3) containing 0.2 g/l yeast extract (Beckton, Dickinson and Co., Franklin Lakes, NJ, USA), 22 mM KH_2_PO_4_, 11 mM K_2_HPO_4_, 0.8 mM MgSO_4_·7H_2_O, 24 mM NaCl, 19 mM NH_4_Cl, 81 µM MnCl_2_·4H_2_O, 6.3 µM FeCl_3_·6H_2_O, and 8.8 µM CaCl_2_·2H_2_O. For all experiments, bacterial cultures and solutions were used at 1×10^7^–5×10^7^ CFU/ml.

### *In vitro* assay for diacetyl generation and screen for inhibitory agents

Diacetyl is produced via acetolactate from pyruvate, which is formed from organic and amino acids such as l-lactate, serine, alanine, glycine, and valine. Therefore, these components were selected as the main substrates for analysis in the present study. Each sweat component–sodium pyruvate, sodium l-lactate (Sigma-Aldrich, St. Louis, MO, USA), serine, alanine, glycine, or valine–was added at a final concentration of 2 mM to semi-synthetic medium containing a single bacterial species. Bacterial suspensions (10 ml) in glass tubes were incubated aerobically at 36°C for 6 or 24 h with shaking.

The inhibitory effects of 156 materials, including plant extracts and cosmetic ingredients, on diacetyl formation were evaluated in *S*. *aureus* and *S. epidermidis*. Sodium l-lactate was added at a final concentration of 2 mM to semi-synthetic medium containing a single bacterial species; 100 µl of plant extract or cosmetic ingredient in 50% (v/v) 1,3-butanediol solution or ethanol, respectively, was added to the solution to obtain final concentrations ranging from 3.0 to 10 mg/ml (plant extracts) or 0.2 mM to 7.2 µM (cosmetic ingredients). The 10-ml solutions were incubated aerobically for 6 h. The solvents–i.e., 50% (v/v) 1,3-butanediol solution or ethanol–used to dissolve the cosmetic materials were used as controls. At the beginning and end of the incubation, bacterial viability was determined by a total viable count analysis on soybean-casein digest plates incubated aerobically at 35°C for 48–72 h.

All sample solutions after the incubation were passed through a 0.20-µm membrane filter to remove bacteria, and levels of each sweat component and of diacetyl in the filtrate were quantitated by high-performance liquid chromatography (HPLC).

### Determination of sweat component and diacetyl levels by HPLC

An LC-VP HPLC system (Shimadzu, Kyoto, Japan) equipped with an LC-20ADVP pump, an SIL-20A autosampler, and an SPD-M10A photodiode array detector was used. The Shimadzu Class VP software was used for automatic integration of the peak area. The separation was performed with a YMC hydrosphere C18 S-5 µm, 4.6×250-mm column (YMC, Kyoto, Japan). For quantitation of each sweat component metabolized by *S. aureus* and *S. epidermidis*, HPLC conditions were as follows: a mobile phase consisting of 20 mM NaH_2_PO_4_ and 0.12 M NaCl; solvent flow of 0.7 ml/min at 30°C; and sample injection into a 20-µl sample loop. Pyruvate, l-lactate, serine, alanine, glycine, and valine were detected at 210 nm and quantitated based on standard curves.

The procedure for diacetyl quantitation was slightly modified from a previously reported method [23]. The reaction mixture was prepared by dissolving 50 mg of 2, 4-dinitrophenylhydrazine (DNPH) in 7 ml methanol and 1 ml of 12 M hydrochloric acid. The solution was transferred to an ice-cold tube, and a reaction solution was prepared by adding 2 ml aniline. To derivatize the diacetyl in the sample, 0.7 ml of the sample was added to 0.7 ml of the reaction solution, and the mixture was incubated for 60 min at 30°C to allow diacetyl-DNPH derivatives to form. The sample solution was then passed through a 0.45-µm membrane filter, and HPLC was used to quantitate the diacetyl levels in the filtrate. Derivatized samples were chromatographically eluted using a mobile phase consisting of aqueous acetonitrile (50∶50, v/v) delivered at 1 ml/min, and injected into a 100-µl sample loop. The flow rate was 1 ml/min at 50°C, and diacetyl-DNPH was detected at 365 nm.

### Metabolic flux analysis

Extra- and intracellular metabolic flux analyses were performed to investigate the suppressive mechanisms of *Glycyrrhiza glabra* root extract (GGR) and α-tocopheryl-l-ascorbate-2-O-phosphate diester potassium salt (EPC-K1) (Senju Pharmaceutical, Osaka, Japan), which indicated high inhibitory effects on diacetyl formation. The metabolic changes by addition of 2-n-heptyl-4-hydroxyquinoline-N-oxide (HQNO) (Enzo Life Sciences, Inc., New York, USA) was also analyzed. *S. aureus* has been detected from the human axillae and feet [16], [24]. We used *S. aureus* as a proxy organism for common human skin bacteria given that the major human skin bacteria are in the genus *Staphylococcus* and *S. aureus* was shown to produce the highest levels of diacetyl in vitro. [U-^13^C] Sodium l-lactate (U-13C3, 98%; Cambridge Isotope Laboratories, Andover, MA, USA) was added at a final concentration of 2 mM to semi-synthetic medium containing a single bacterial species; 3.0 mg/ml GGR or 7.2 µM EPC-K1 in 50% (v/v) 1,3-butanediol solution or 77 nM HQNO in ethanol was then added to the solution. Sample solutions (110 ml) were incubated aerobically at 36°C for 0, 3, 5, and 7 h with shaking. Levels of the extracellular metabolites l-lactate, pyruvate, diacetyl, and acetoin in 10-ml samples were determined by HPLC as described above.

To investigate the time course of changes in intracellular metabolite composition after the addition of EPC-K1, GGR, and HQNO, 100-ml samples obtained at each time point were passed through a 0.40-µm pore size filter (Millipore, Billerica, MA, USA). Residual cells on the filter were washed twice with 10 ml Milli-Q water, and the filter was soaked in 2 ml methanol containing internal standards (H3304-1002; Human Metabolome Technologies, Inc., Tsuruoka, Japan) and sonicated for 30 s at 28 kHz to disrupt cells and collect intracellular metabolites, which were then washed with 1 ml methanol. A total of 3 ml of cell extract was transferred to new tubes and concentrated to 1.5 ml by centrifugal evaporation; 1.5 ml chloroform and 0.9 ml Milli-Q water were added to the solution, which was centrifuged at 2300×*g* for 5 min at 4°C. The upper aqueous layer was filtered by centrifugation through a 5 kDa-cutoff filter (Millipore) at 9100×*g* and 4°C for 120 min to remove proteins. The filtrate was concentrated by centrifugation to 50 µl for capillary electrophoresis-mass spectrometry (CE-MS) analysis, which was carried out using a CE system equipped with a 6210 time-of-flight mass spectrometer, 1100 isocratic HPLC pump, G1603A CE-MS adapter kit, and G1607A CE electrospray ionization MS sprayer kit (all from Agilent Technologies, Santa Clara, CA, USA). Metabolites were analyzed using a fused silica capillary (50 µm inner diameter [i.d.]×80 cm total length) in commercial electrophoresis buffer containing electrolytes (solutions H3301-1001 and H3302-1021 for cation and anion analyses, respectively; Human Metabolome Technologies, Inc.). The sample was injected at a pressure of 50 mbar for 10 s (∼10 nl) for cation and 25 s (∼25 nl) for anion analyses. The spectrometer scanned from m/z 50 to 1000. Other conditions were as previously described [25]–[27]. Peaks were extracted using MasterHands automatic integration software (Keio University, Tsuruoka, Japan) to obtain peak information, including m/z, migration time for CE-MS measurements, and area [28]. The annotation of detected peaks was performed based on m/z and migration time (MT) in CE of standard chemicals for each of the monoisotopic ions, with a deviation of ±10 ppm for m/z and ±0.5 min for MT. The annotation of isotopic ions was performed based on calculated m/z values and the above MT values for each target compound, whose concentrations were calculated based on the value (in µM) detected for corresponding standard chemicals.

### Fatty acid analysis

An aliquot of *S. aureus* culture was added to semi-synthetic medium supplemented with sodium l-lactate and 7.2 µM EPC-K1 in 50% (v/v) 1,3-butanediol solution. After incubating in aerobic conditions for 5 h at 36°C, the bacterial solution was centrifuged at 6900×*g* for 15 min at 4°C, washed twice with 0.85% (w/v) saline, and the bacterial pellet (dry weight) was retained. Methylated fatty acid levels in bacteria (2.5 ml) were determined using a kit (Nacalai Tesque, Kyoto, Japan) according to the manufacturer’s instructions. An internal standard (dimethyl phthalate) was added to the extracted sample solution, and the liquid was evaporated from the mixture under a stream of nitrogen and the residue was dissolved in 100 µl n-hexane. Identification and quantitation of fatty acids in the sample were performed by gas chromatography (GC)-MS (Agilent Technologies) and GC with a flame ionization detector (GC-FID) at 250°C with a DB-23 capillary column (30 m×0.25 mm i.d. with 0.25-µm film thickness; J & W Scientific Inc., Folsom, CA, USA), respectively. Helium was used as the carrier gas at a flow rate of 0.9 ml/min. Samples (5 µl) were injected at 260°C with a split ratio of 5. Pulsed split injection was used with an injector pressure of 40 psi for 1 min. The oven temperature was ramped from 60°C to 150°C at a rate of 6.5°C/min, then to 180°C at a rate of 1°C/min, and finally to 230°C at a rate of 5°C/min, where it was held for 5 min. Mass spectra were obtained in electron-impact mode (70 eV) with transfer and source temperatures of 240°C and 230°C, respectively. Fatty acids in the samples were identified by matching mass spectral fragmentation patterns with those stored in the National Institute of Standards and Technology 08 GC-MS data system [29], an automated mass spectral deconvolution and identification system [30], and retention times were compared to those of available authentic standards. Quantitation values for each of the 15 species of fatty acids in the samples were obtained using the standard curve of methyl palmitate, and the total amount of fatty acids in the cells was calculated from the sum of the quantitation values.

## Results

### Identification of human skin bacteria and sweat components responsible for diacetyl generation

To find resident skin bacteria capable of diacetyl formation, nine strains of human skin bacteria (*Staphylococcus* and *Corynebacterium* spp.) with the relevant metabolic pathways were screened based on the Kyoto Encyclopedia of Genes and Genomes (KEGG) pathway database [31]. Using sodium pyruvate as the substrate, diacetyl was produced by *S*. *aureus* and *S. epidermidis* (Table 1), with the former having the highest capacity for diacetyl formation. *S*. *capitis*, *S*. *haemolyticus*, and *Corynebacterium* species were unable to produce diacetyl under the conditions of our experiments.

**Table 1:** Diacetyl and acetoin formation by *Staphylococcus* and *Corynebacterium* spp.

Bacterial strain	Mean ± S.D. (µM)	
	Diacetyl	Acetoin
*Staphylococcus aureus* NBRC13276	4.82±0.30	261.32±24.31
*Staphylococcus epidermidis* IAM1296	2.67±0.09	127.85±5.16
*Staphylococcus hominis* ATCC35982	0.20±0.04	23.49±1.04
*Staphylococcus capitis* ATCC2784	<0.12	<17.00
*Staphylococcus haemolyticus* ATCC29970	<0.12	<17.00
*Corynebacterium striatum* ATCC694	<0.12	<17.00
*Corynebacterium jeikeium* ATCC43734	<0.12	<17.00
*Corynebacterium xerosis* NBRC12684	<0.12	<17.00
*Corynebacterium minutissumum* ATCC23348	<0.12	<17.00

Bacterial cultures supplemented with 2 mM pyruvate were incubated for 24 h. Results are given as means ± standard deviation of three independent experiments. Detection limits of diacetyl and acetoin were 0.12 µM and 17 µM, respectively.

**Figure 1:**
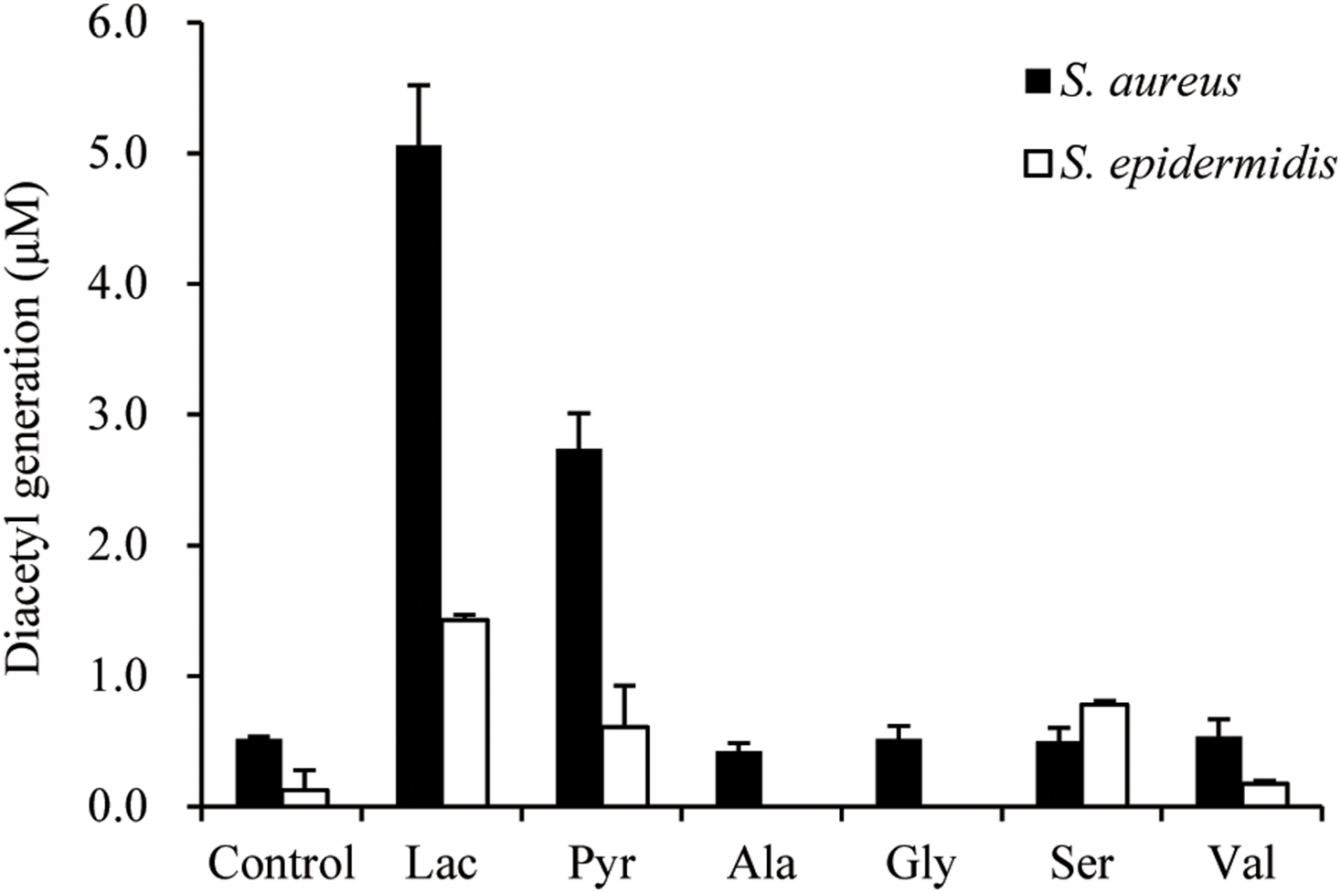
Evaluation of sweat components as precursors of diacetyl formation. Abbreviations are: Lac, l-lactate; Pyr, pyruvate; Ala, alanine; Gly, glycine; Ser, serine; Val, valine. Bacterial cultures supplemented with 2 mM each substrate were incubated for 6 h. Control samples were treated with sterilized water as substrate. The error bars indicate the standard deviation of three independent experiments.

*S*. *aureus* and *S. epidermidis* were used to evaluate the ability of sweat components (pyruvate, l-lactate, serine, alanine, glycine, and valine) to serve as precursors for diacetyl generation. The largest amounts of diacetyl were generated by *S*. *aureus* and *S. epidermidis* with l-lactate as the substrate (Figure 1).

### Inhibitory effect of cosmetic materials on diacetyl formation

**Figure 2:**
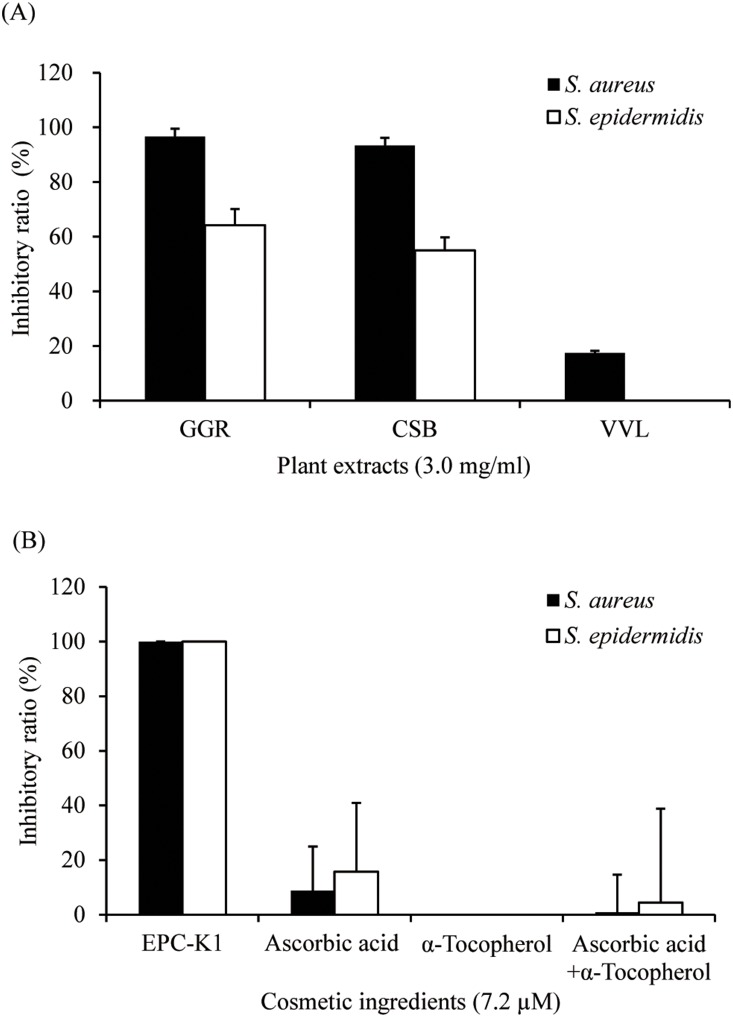
Inhibitory effects of plant extracts (A) and cosmetic ingredients (B) on diacetyl formation. Inhibition by plant extracts and cosmetic ingredients was observed at 3.0 mg/ml and 7.2 µM, respectively. Each sample was incubated for 6 h. Abbreviations are: GGR, *Glycyrrhiza glabra* root; CSB, *Cinchona succirubra* bark; VVL, *Vitis vinifera* leaf; EPC-K1, α-tocopheryl-l-ascorbate-2-O-phosphate diester potassium salt. The error bars indicate the standard deviation of three independent samples. The inhibitory ratio (%) was calculated using the following equation: % = [(1−amount of diacetyl formation in each material)/(amount of diacetyl formation in the control)]×100.

**Figure 3:**
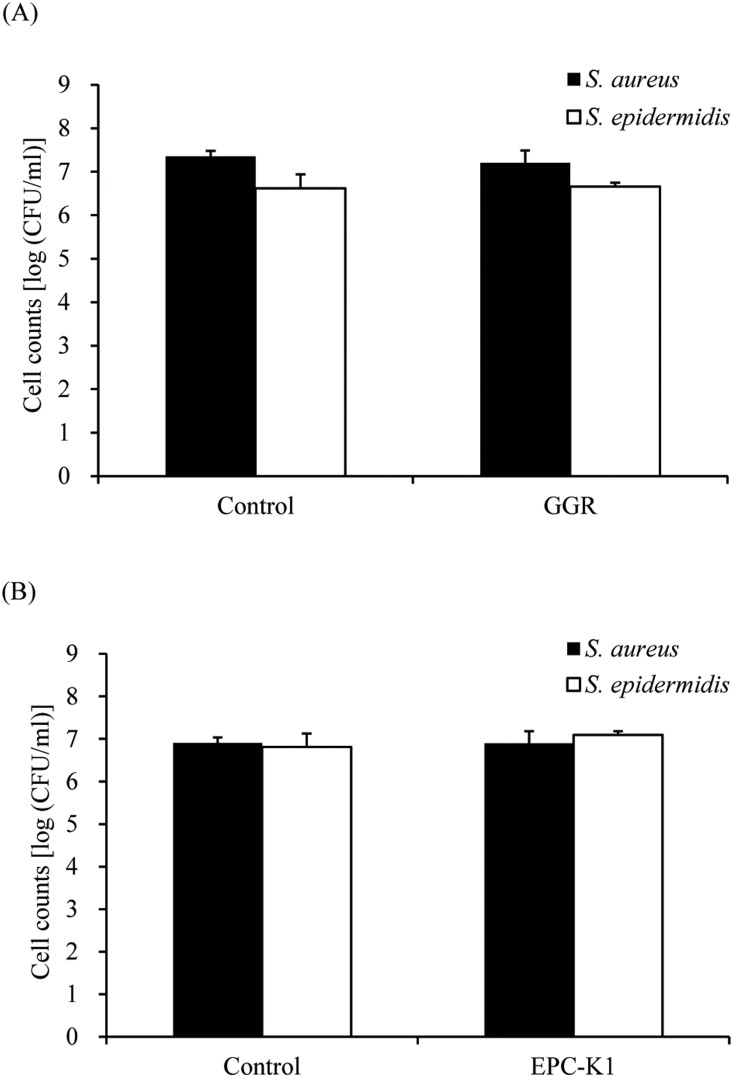
Cell counts of *S. aureus* and *S. epidermidis* incubated with 3.0 **mg/ml GGR (A) and 7.2** µ**M EPC-K1 (B).** Control samples were treated with 50% (v/v) 1,3-butanediol. Viable cells were counted after a 6-h incubation. Results are shown as means ± standard deviation of three independent experiments.

Over 100 plant extracts and 50 cosmetic ingredients were assessed to find effective inhibitors of diacetyl generation by *S*. *aureus* and *S. epidermidis*; GGR and EPC-K1 were active against both bacteria at lower concentrations and were selected for experiments. Diacetyl formation was markedly suppressed by 3.0 mg/ml GGR (Figure 2A), and fully inhibited by 7.2 µM EPC-K1, while ascorbic acid, α-tocopherol, and mixtures of these compounds had no effect (Figure 2B). Furthermore, Figure 3 shows that GGR and EPC-K1 did not exhibit bactericidal activity.

### Mechanisms of GGR and EPC-K1 suppression of diacetyl formation

**Figure 4:**
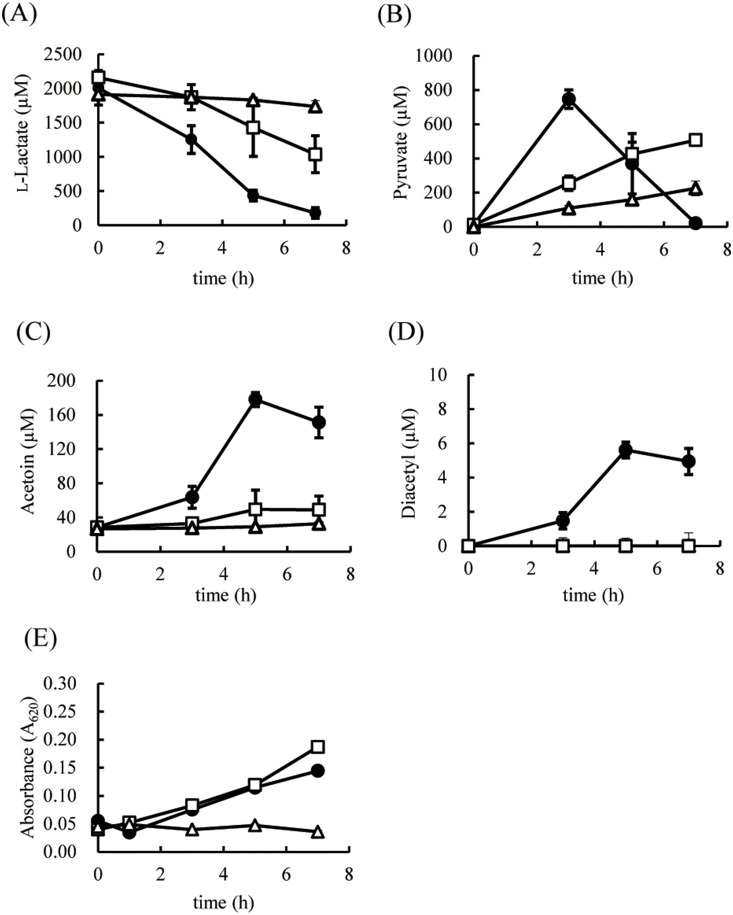
Time course of changes in extracellular metabolite concentrations (A–D) and growth curves (E) of *S. aureus* following treatment with 3.0 **mg/ml GGR (Δ) and 7.2** µ**M EPC-K1 (□).** Shown are concentrations of l-lactate (A), pyruvate (B), acetoin (C), and diacetyl (D). Control samples (•) were treated with 1,3-butanediol. Results are shown as means ± standard deviation of three independent experiments.

To elucidate the mechanism of GGR- and EPC-K1-mediated inhibition, extracellular and intracellular metabolic flux analyses were performed in *S. aureus* by measuring changes in the levels of extracellular metabolites (l-lactate, pyruvate, diacetyl, and acetoin) by HPLC. *S. aureus* used l-lactate gradually, consuming 90% of the substrate by 7 h (Figure 4A); extracellular pyruvate accumulation was observed up to 3 h, after which it was taken up by the cells (Figure 4B). Concentrations of excreted diacetyl and acetoin reached maximum values at 5 h (Figure 4C, D). In contrast, l-lactate was a poor substrate in the presence of GGR, with reduced pyruvate formation at 7 h (Figure 4A, B). EPC-K1 also decreased the rate of l-lactate utilization by *S. aureus*, with 50% (1 mM) of the substrate remaining after 7 h (Figure 4A), while pyruvate production slowly increased compared to the control (Figure 4B). Diacetyl formation by *S. aureus* was not detected after 7 h following treatment with either GGR or EPC-K1, which also suppressed the generation of acetoin (Figure 4C, D). The growth rate increased slightly for the control (Figure 4E). On the other hand, cell growth was suppressed by GGR, indicating bacteriostasis, but was essentially unaffected by EPC-K1 (Figure 4E).

**Figure 5:**
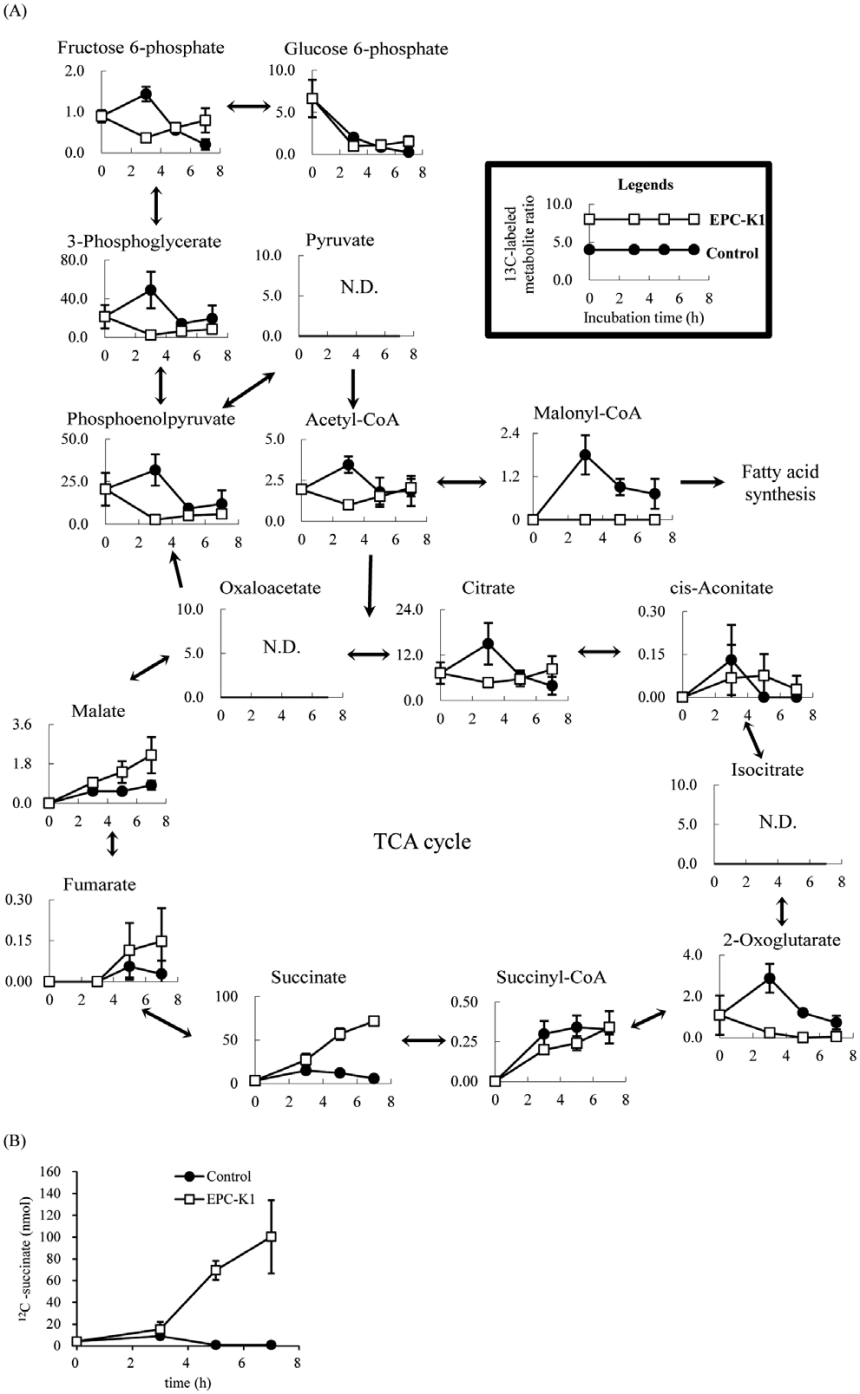
Flux profiling of ^13^C-labeled glycolytic and tricarboxylic acid (TCA) cycle metabolites (A) and ^12^C-succinate (B) of *S. aureus* following treatment with 7.2 µ**M EPC-K1 (□) or 1,3-butanediol as a control (**•**).** ^13^C-labeled metabolite ratio was calculated using the following equation: (^13^C-labeled metabolite concentration at each incubation time/total quantitation values of ^13^C-labeled metabolite concentration at each incubation time)×1000. ^13^C-labeled metabolites indicate the total quantitation values from *M*_1_ to *M*_i_, where *M*_i_ represents the isotopomer fraction for each metabolite in which *i* ^13^C atoms are incorporated. Results are shown as means ± standard deviation of three independent experiments. N.D., not detected.

The effects of GGR and EPC-K1 were also evaluated by measuring changes in 77 ^13^C-labeled intracellular metabolites in *S. aureus* by CE-MS. Following treatment with GGR, the total amount of these metabolites was lower than that in the control (Figure S1), demonstrating that GGR-induced inhibition of diacetyl production is due to suppression of l-lactate uptake. In contrast, 7 h after the addition of EPC-K1, the metabolite ratio of ^13^C-labeled succinate–that is, (^13^C-labeled metabolite concentration at each incubation time/total quantitation values of ^13^C-labeled metabolite concentration at each incubation time)×1000–in *S. aureus* cells was about 12-fold higher than that in the control (Figure 5A). In addition, ^12^C-succinate, which was not derived from the labeled substrate, accumulated in cells over time to a value 120-fold higher than in the control after 7 h (Figure 5B). While fumarate and malate also accumulated in the cells, the formation of malonyl-CoA, an essential intermediate in fatty acid synthesis, was suppressed by EPC-K1 (Figure 5A). Using total viable counts after 5 h of incubation and energy charge (EC; [ATP+0.5 ADP]/[ATP+ADP+AMP]), which ranges in value from 0 (all AMP) to 1 (all ATP) and is typically between 0.80 and 0.95 [32]–[34], as an index of energy status, we found no difference for *S. aureus* between control and GGR- or EPC-K1-treated cells (Figure S2).

### Metabolic flux changes of *S. aureus* following HQNO treatment

**Figure 6:**
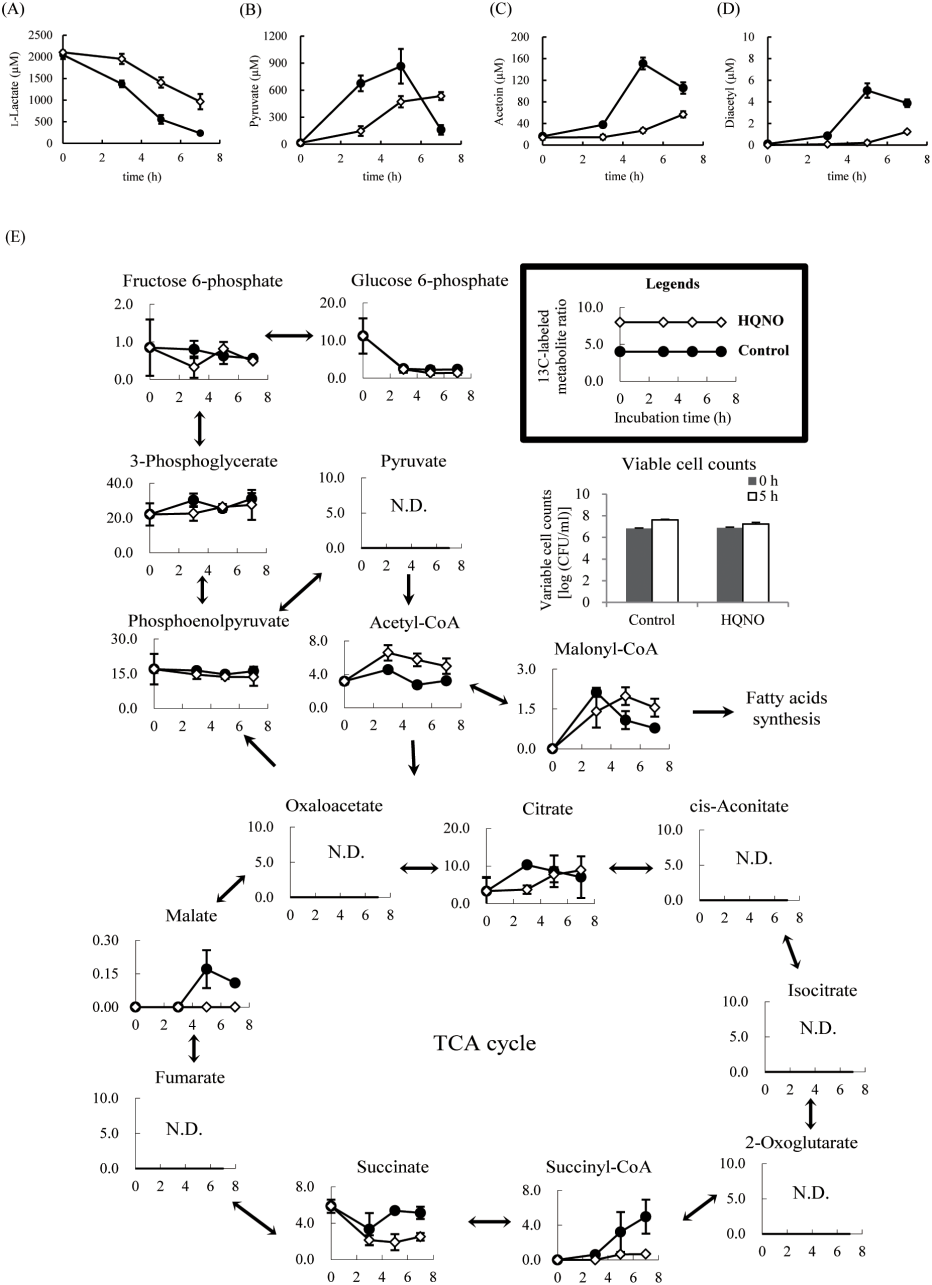
Time course of changes in extracellular metabolite (A–D) and ^13^C-labeled intracellular metabolite (E) concentrations of *S. aureus* following treatment with 77 **nM HQNO (⋄).** Shown are the concentrations of l-lactate (A), pyruvate (B), acetoin (C), and diacetyl (D). Control samples (•) were treated with EtOH. ^13^C-labeled metabolite ratio was calculated using the following equation: (^13^C-labeled metabolite concentration at each incubation time/total quantitation values of ^13^C-labeled metabolite concentration at each incubation time)×1000. ^13^C-labeled metabolites indicate the total quantitation values from *M*_1_ to *M*_i_, where *M*_i_ represents the isotopomer fraction for each metabolite in which *i* ^13^C atoms are incorporated. Results are shown as means ± standard deviation of three independent experiments. N.D., not detected.

To confirm that the suppression of diacetyl formation by EPC-K1 is due to inhibition of succinate metabolism, changes in diacetyl formation and intracellular metabolite accumulation were assessed by a metabolic flux analysis of *S. aureus* using HQNO, a menaquinone analog and succinate dehydrogenase (SDH; E.C. 1.3.99.1) inhibitor. Under the present growth conditions, *S. aureus* utilized 50% (1 mM) of l-lactate by HQNO, just as observed with EPC-K1 treatment (Figure 6A). However, unlike EPC-K1, HQNO did not fully inhibit the production of diacetyl and acetoin (Figure 6C, D). The formation of malate and fumarate was fully suppressed and that of succinate was down-regulated from 3-h incubation (Figure 6E). Moreover, changes in other metabolites–including malonyl-CoA–by HQNO differed from those induced by EPC-K1 (Figure 6E).

### Changes in fatty acid levels and components induced by EPC-K1

**Figure 7:**
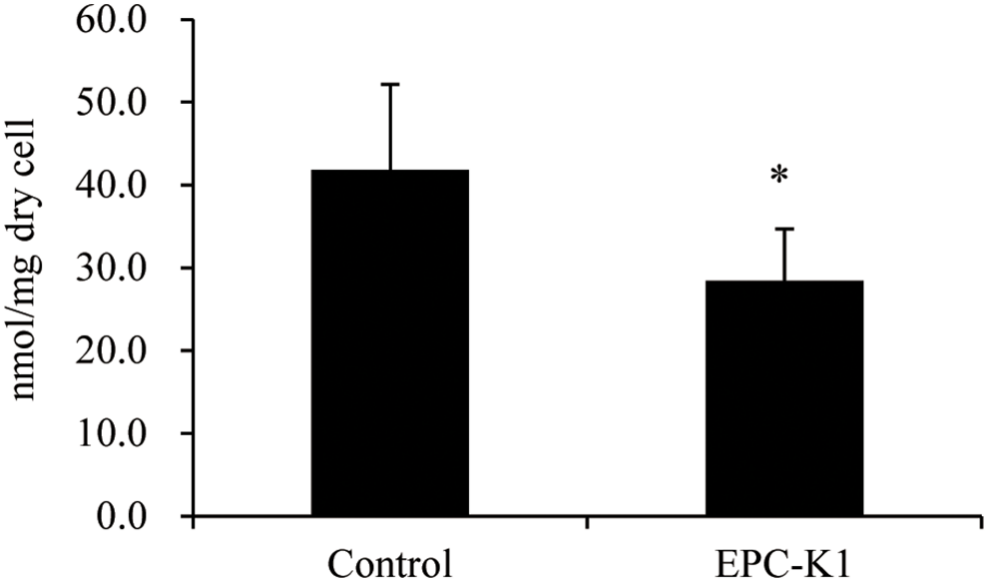
Suppression of fatty acid biosynthesis of *S. aureus* following EPC-K1 application. The total amounts of fatty acids in the cells were calculated using the total quantitation values of fatty acids detected by gas chromatography-mass spectrometry. Results are shown as means ± standard deviation (n = 6). *P<0.05 vs. 1,3-butanediol (control) (Student’s t-test).

To assess the changes in fatty acid levels and profiles induced by EPC-K1 treatment, the total amount and composition of fatty acids in *S. aureus* cells was calculated from measurements of 15 fatty acids by GC-FID and GC-MS, and the levels of fatty acids were compared against a standard curve generated using palmitic acid. The fatty acids in *S. aureus* control cells represented 41.8 nmol/mg dry cells. Following treatment with EPC-K1, fatty acid levels decreased to 28.5 nmol/mg dry cells (Figure 7). We also analyzed the fatty acid composition of *S. aureus* following the addition of EPC-K1 (Table 2). The composition (percentage) of branched-chain fatty acids in the cells decreased from 82.2% to 67.3% by EPC-K1 treatment; in particular, the ratios of anteiso 15∶0 and anteiso 17∶0 were both markedly reduced, by 10%, compared to the control.

**Table 2:** Fatty acid profiles of *S. aureus* following EPC-K1 application.

Fatty acid	Composition (%)^a^	
	Control	EPC-K1
C14∶0 anteiso	1.15±0.20	2.03±0.52
C14∶0	0.61±0.24	1.49±0.62
C15∶0 iso	4.49±0.19	6.11±0.44
C15∶0 anteiso	41.07±0.99	30.11±2.66
C16∶0 iso	2.40±0.40	2.58±0.70
C16∶0	1.59±0.26	2.69±0.61
C17∶0 iso	3.39±0.28	3.13±0.52
C17∶0 anteiso	21.33±1.05	10.18±0.92
C18∶0 iso	1.46±0.17	1.97±0.60
C18∶0	6.33±0.93	8.39±0.93
C19∶0	1.95±0.20	3.54±0.43
C19∶0 iso	6.18±0.62	9.27±0.84
C20∶0 iso	0.75±0.21	1.90±0.54
C20∶0	6.42±0.74	14.10±2.58
C21∶0	0.87±0.30	2.51±0.73
Straight-chain	17.8	32.7
Branched-chain	82.2	67.3

a *S. aureus* cells were grown in semi-synthetic medium supplemented with 2 mM sodium l-lactate and 7.2 µM EPC-K1 for 5 h. 1,3-Butanediol was used as a control. Results are given as means ± standard deviation of six independent experiments.

## Discussion

This study shows that regulation of the *Staphylococcus* spp. metabolic route from lactate to diacetyl inhibits the generation of diacetyl. Specifically, we demonstrated that GGR and EPC-K1 effectively inhibited diacetyl formation by two distinctive metabolic regulation mechanisms.

The human skin harbors various species of bacteria. High population densities of *Staphylococcus* and aerobic coryneform bacteria are present on human feet, but anaerobic (e.g., *Propionibacterium*) and gram-negative bacteria are not highly represented among human feet microflora [14]. The major axillary microflora includes aerobic coryneforms, propionibacteria, staphylococci, and micrococci [24], [35], and with the exception of micrococci, all of these are also prevalent in occiput and toe web spaces in healthy humans [36]. In this study, resident skin bacteria that are capable of diacetyl formation were screened. *Staphylococcus* and *Corynebacterium* spp. were used as representative human skin bacteria after an examination of the KEGG database revealed that these genera encode metabolic enzymes related to diacetyl formation [31], and *S. aureus* and *S. epidermidis* were identified as key commensal species for this process (Table 1). In contrast, *Corynebacterium* spp. were incapable of diacetyl formation under our experimental conditions (Table 1). These results suggest that *S. aureus* and *S. epidermidis* play important roles in the generation of diacetyl in human skin.

Diacetyl is produced via acetolactate from pyruvate, which is formed from organic and amino acids such as l-lactate, serine, alanine, glycine, and valine. Moreover, pyruvate and its precursors are abundant in human axillary sweat (Table S1); indeed, l-lactate, pyruvate, and free amino acids were detected in samples collected from the soles of male feet by high-resolution nuclear magnetic resonance [37]. Clear precursor-product relationships were demonstrated between pyruvate and its precursors, which generated significant levels of diacetyl by bacterial metabolism of the l-lactate substrate (Table 2). *Staphylococcus* and *Propionibacterium* spp. metabolize lactate to form short-chain fatty acids as fermentation products [38]. We confirmed that human sweat contains higher amounts of l-lactate than other organic and amino acids (Table S1). Thus, the present results support previous findings that the generation of diacetyl on human skin is mainly due to lactate metabolism by *S. aureus* and *S. epidermidis*.

This biotransformation assay demonstrated that GGR and EPC-K1 have a high capacity to suppress diacetyl formation (Figure 2). GGR contains dipotassium glycyrrhizate, which is reported to be an anti-inflammatory agent [39]; however, dipotassium glycyrrhizate in itself did not inhibit diacetyl formation (data not shown). Experiments are underway to identify the chemical compound in GGR that is responsible for its inhibitory effects. EPC-K1 is a phosphate diester of α-tocopherol and l-ascorbic acid–two endogenous compounds that independently protect against oxidative stress-induced injury [40]–which also functions as a hydroxyl radical scavenger [41]. However, neither α-tocopherol nor l-ascorbic acid alone or in combination affected diacetyl formation (Figure 2B), suggesting that the inhibitory mechanism of EPC-K1 on diacetyl formation is not due to its antioxidant properties. Moreover, no bactericidal effects were associated with GGR and EPC-K1 (Figure 3). These results indicate that the decrease in viable bacterial counts did not directly cause the inhibitory effects of these compounds on the generation of diacetyl. Alternatively, we suggest that the inhibitory effects of these two compounds are due to the suppression of metabolic processes that produce diacetyl, including the assimilation of l-lactate.

Metabolites are replaced over time with newly synthesized compounds; as such, information from metabolic flux analyses is necessary to determine the mechanism by which GGR and EPC-K1 inhibit bacterial metabolism without exerting bactericidal effects. Using U-^13^C-l-lactate as a substrate, the metabolism of this carbon source via intracellular metabolic pathways can be established based on the labeling patterns of resultant cellular compounds. Although both GGR and EPC-K1 suppressed diacetyl formation by *S. aureus*, the rates of l-lactate metabolism in the presence of these two agents differed greatly (Figure 4A). Membrane-bound l-lactate dehydrogenase (enzyme class [E.C.] 1.1.1.27) catalyzes the oxidation of l-lactate to pyruvate at the bacterial cell surface [42], [43]. The findings that extracellular l-lactate was an inferior substrate in the presence of GGR, and that the total amount of ^13^C-labeled intracellular metabolites was lower than in the control demonstrate that GGR inhibits the bacterial metabolism of extracellular l-lactate (Figure S1). Following the addition of GGR, the viable count of *S. aureus* cells did not change (Figure 3A), and the optical density values at 620 nm (OD_620_) were maintained at 0.04 from 0 to 7 h of incubation, reflecting a density of 1×10^7^–2×10^7^ CFU/ml (Figure 4E). Thus, *S. aureus* cells did not die upon treatment of GGR; on the other hand, the cells were not in a proliferative state. Thus, these results indicate that the inhibition of l-lactate uptake by GGR suppressed the growth of *S. aureus*. On the other hand, changes in gene expression in the bacteria could also occur in response to changes in the growth environments, such as those involved in growth rate, nutritional stress, and cell damage [44]–[46]. Considering that the cell viability and EC of *S. aureus* were unaffected while the cell growth rate was suppressed by GGR (Figure 4E and S2), changes in the expression of the gene(s) involved in cell growth might be induced to facilitate survival and/or protect the bacteria from the stress imposed by GGR. Therefore, the expression of genes involved in l-lactate dehydrogenase might change upon exposure to GGR, which could inhibit l-lactate incorporation. This hypothesis should be examined in future studies.

Although only half of the l-lactate as extracellular substrate was consumed by *S. aureus* after 7 h, EPC-K1 suppressed diacetyl formation as efficiently as GGR (Figure 4A, D). Furthermore, there were no differences in growth rates between EPC-K1-treated and control cells (Figure 4E). Since EPC-K1 was expected to affect the intracellular metabolic pathways, changes in 77 major bacterial metabolites, with a focus on central metabolic pathways, were investigated in detail. The intracellular accumulation of ^12^C-succinate was found to be greater than that of ^13^C-succinate (Figure 5), suggesting that in addition to ^13^C-labeled substrates, the original^ 12^C- intracellular metabolites were shunted to the tricarboxylic acid cycle. Nonetheless, the production of ^13^C-labeled malonyl-CoA was markedly inhibited by the addition of EPC-K1 (Figure 5A), as was ^12^C-malonyl-CoA (data not shown). The glycolysis and metabolism of 20 different amino acids involved in central metabolic pathways were unaltered by EPC-K1 treatment (data not shown). SDH is part of the nonoxidative branch of the tricarboxylic acid cycle and is directly linked to the respiratory chain. This enzyme complex catalyzes the oxidation of succinate to fumarate along with the reduction of menaquinone to menaquinol. This enzymatic activity requires three subunits: membrane-bound cytochrome *b*_558_ (SdhC), a flavoprotein containing a flavin adenine dinucleotide-binding site (SdhA), and an iron-sulfur protein with a binding region signature of the 4Fe-4S type (SdhB) [47]–[49]. Changes in extracellular metabolite accumulation were observed using deletion mutants of SDH genes (*sdh*CAB and SA113Δ*sdh*) in *S. aureus* SA113 [50]. According to this report, succinate accumulated outside SA113Δ*sdh* cells in a chemically defined medium, while acetoin was produced by SA113Δ*sdh* as well as wild-type cells. Therefore, we analyzed the changes in diacetyl formation and intracellular metabolite accumulation using HQNO, a menaquinone analog and SDH inhibitor. Under our experimental conditions, HQNO down-regulated the formation of succinate, malate, and fumarate by *S. aureus* (Figure 6E). On the other hand, HQNO did not fully inhibit the production of diacetyl and malonyl-CoA, in contrast to EPC-K1 treatment (Figure 6D, E). Thus, these results suggest that inhibiting malonyl-CoA as well as succinate metabolism would be a key factor in the suppression of diacetyl formation by this compound.

Malonyl-CoA is a global regulator of lipid homeostasis in gram-positive bacteria [51] and an essential intermediate in fatty acid synthesis, functioning as a chain extender of fatty acids that are essential membrane components and sources of metabolic energy [52]–[54]. The percentage of unesterified total fatty acids in aerobically grown *S. aureus* was less than 0.12%, and 87% of total fatty acids were present as glyco- and phospholipids, the major lipid types in the cell membrane [55]. The branched-chain membrane fatty acids in bacteria are produced by malonyl-CoA and α-keto acids as primers [53], [56]. Given the effect of EPC-K1 on malonyl-CoA synthesis, changes in fatty acid levels and fatty acid profiles were expected in the presence of EPC-K1; indeed, addition of EPC-K1 resulted in a 32% decrease in fatty acid levels (Figure 7) and reduced the branched-chain membrane fatty acid composition (Table 2). If cell membrane synthesis had been completely inhibited, the bacteria would not retain their original form. However, the shape and size of *S. aureus* cells were markedly unaffected by EPC-K1 treatment based on observations with a scanning electron microscope (Figure S3). In addition, EPC-K1 did not have a bactericidal effect and does not contain long-chained fatty acids or possess a similar structure to such impurities. Therefore, we believe that the decrease in the amount (nmol/mg dry cells) of fatty acids was not due to an increase in the dry weight caused by dead *S. aureus* cells and non-cellular material. Thus, *S. aureus* cells treated with EPC-K1 appeared to have a low level of metabolic activity with respect to fatty acid synthesis, and the cell wall thickness decreased at the nanometer-level, but the cells nonetheless appeared to be alive because their shapes were maintained. Changes in cell wall thickness require further confirmation with transmission electron microscopy. Overall, our results suggest that suppression of malonyl-CoA synthesis by EPC-K1 down-regulated fatty acid synthesis and changed the fatty acid profile of the cell membrane.

**Figure 8:**
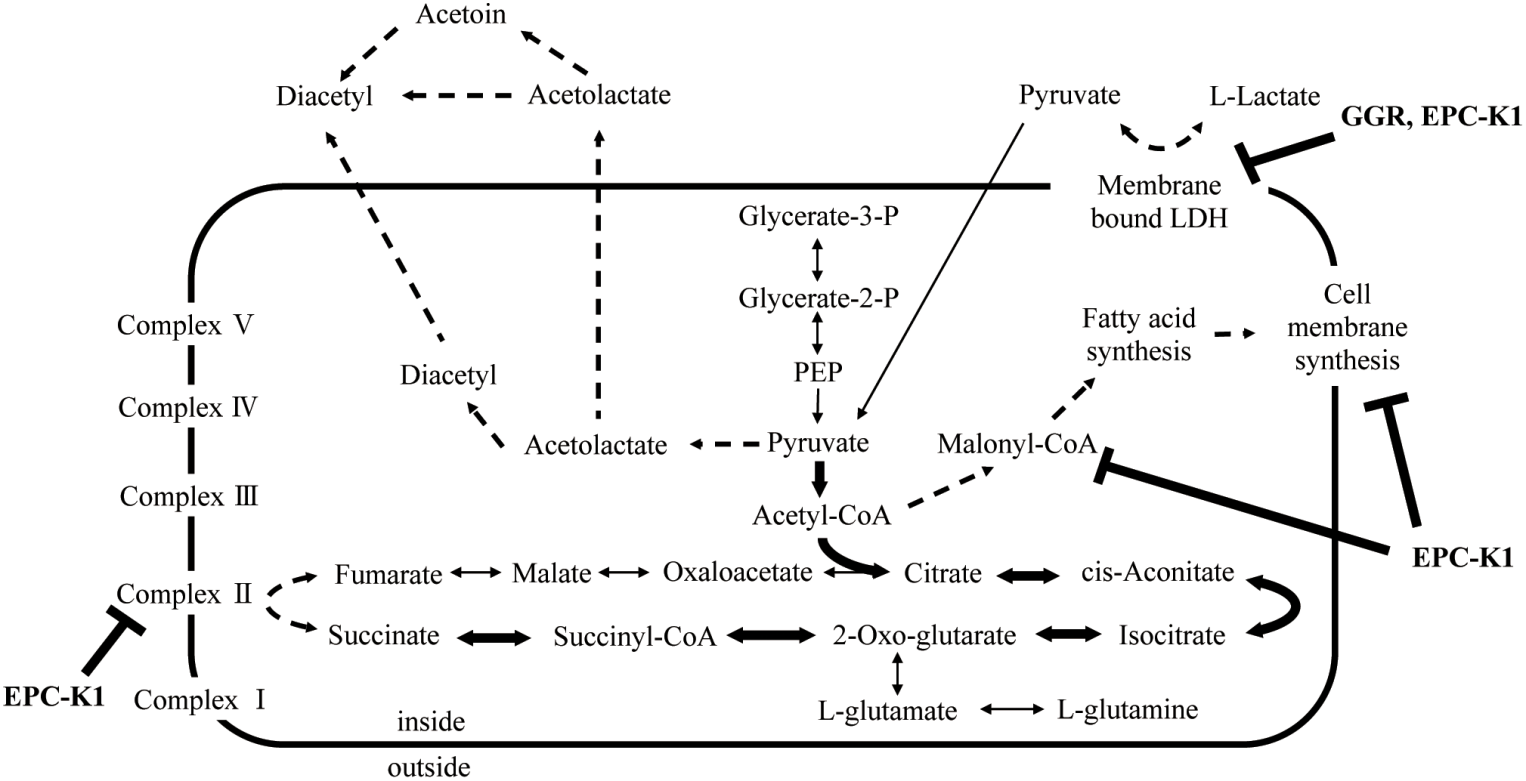
Schematic representation of observed metabolic flow of bacterial metabolism and steps affected by GGR and EPC-K1 addition. Dashed and bold arrows indicate the down- and up-regulation of bacterial metabolism, respectively.

Total viable count and EC were unaffected by EPC-K1 treatment (Figure S2), indicating that reduction in general bacterial activity was not responsible for the observed suppression of diacetyl formation. The total amount of isotopomer (from *m*_1_ to the fully labeled form) for each intracellular metabolite indicated that carbon from [U-^13^C] l-lactate is shunted to the citrate cycle in the presence of EPC-K1 (data not shown). If EPC-K1 completely inhibited succinate metabolism and malonyl-CoA formation, the bacteria would be unable to produce the energy necessary for growth, and cell death would result. Thus, EPC-K1 likely does not completely inhibit, but instead only partly affects, succinate and malonyl-CoA metabolism. As a result, metabolic flow to the citrate cycle would likely increase significantly in order to maintain *S. aureus* growth (Figure 8). Therefore, EPC-K1 would suppress diacetyl formation by regulating the rate of pyruvate-acetolactate-diacetyl transformation.

The present work shows that diacetyl is principally produced by the breakdown of l-lactate by *S. aureus* and *S. epidermidis* and that GGR and EPC-K1 suppress only specific metabolic pathways. It was also demonstrated that the suppression of diacetyl formation is due to metabolic changes in bacteria induced by GGR and EPC-K1. However, further investigation is required to determine the specific sites of inhibition. These results provide novel insight into the mechanism and regulation of diacetyl production on human skin; strategies for controlling this process would be useful to both the cosmetic and food science industries. We believe that a combination of agents that suppress bacterial metabolism (such as GGR and EPC-K1) and those that have antibacterial properties will have a longer-lasting effect than either of these alone, because odor generation is strongly suppressed at two independent steps. These findings are valuable for individuals experiencing psychological stress due to their unpleasant body odor.

## Supporting Information

Figure S1 **Total amount of 13C-labeled intracellular metabolites after addition of 3.0** **mg/ml GGR.** 13C-labeled metabolites indicate the total quantitation values from M1 to Mi, where Mi represents the isotopomer fraction for each metabolite in which i 13C atoms are incorporated. 1,3-Butanediol was used as a control. Results are shown as means ± standard deviation of three independent experiments.
(AI) Click here for additional data file.

Figure S2 **Changes in viable bacterial counts (A, C) and energy charge (B, D) following the addition of 3.0** **mg/ml GGR (A, B) and 7.2** µ**M EPC-K1 (C, D).** Viable cells were counted after a 5-h incubation. The energy charge was calculated using the total quantitation values of 12C and 13C-labeled metabolites. 1,3-Butanediol was used as a control. Results are shown as means ± standard deviation of three independent experiments.
(AI) Click here for additional data file.

Figure S3 **Scanning electron micrographs of S. aureus cells following treatment with 7.2** µ**M EPC-K1 (A) and 50% (v/v) 1,3-butanediol (B) as a control.** Each sample was treated after incubation for 7 h. Scale bars indicate 10 µm.
(AI) Click here for additional data file.

Table S1 **Compositions of organic acids and amino acids in human axillary sweat of males.**
(DOCX)
