# Supplementary material for: Suppression of Microbial Metabolic Pathways Inhibits the Generation of the Human Body Odor Component Diacetyl by Staphylococcus spp
Source: PLoS One. 2014 Nov 12;9(11):e111833. doi: 10.1371/journal.pone.0111833 (PMC4229079; doi:10.1371/journal.pone.0111833)
Supplement: Table S1 — Compositions of organic acids and amino acids in human axillary sweat of males. (DOCX) [file pone.0111833.s004.docx]

**SUPPORTING INFORMATION**

Supplementary Table 1. Compositions of organic acids and amino acids in human axillary sweat of males.

| Metabolites | Composition (%)*^a^* |
| --- | --- |
| Lactate | 87.9 ± 7.73 |
| Pyruvate | 3.00 ± 0.57 |
| Serine | 2.53 ± 0.27 |
| Glycine | 1.52 ± 0.16 |
| Alanine | 0.91 ± 0.11 |
| Citruline | 0.86 ± 0.09 |
| Threonine | 0.53 ± 0.06 |
| Histidine | 0.53 ± 0.06 |
| Valine | 0.22 ± 0.02 |
| Glutamate | 0.20 ± 0.03 |
| Proline | 0.19 ± 0.02 |
| Arginine | 0.17 ± 0.02 |
| Others | 1.41 ± 0.13 |

*^a^* Human sweat was directly collected from the body site after exercise for 15 min. A total of 67 ionic metabolites in the sweat were quantitated by CE-TOFMS. Results are presented as mean ± standard deviation of five independent experiments.
